# Phenotypic Transformation Affects Associative Learning in the Desert Locust

**DOI:** 10.1016/j.cub.2013.10.016

**Published:** 2013-12-02

**Authors:** Patrício M.V. Simões, Jeremy E. Niven, Swidbert R. Ott

**Affiliations:** 1 Department of Zoology, University of Cambridge, Downing Street, Cambridge CB1 3EJ, UK; 2 International Neuroscience Doctoral Programme, Champalimaud Neuroscience Programme/Instituto Gulbenkian de Ciência, 1400-038 Lisbon, Portugal; 3 School of Life Sciences and Centre for Computational Neuroscience and Robotics, University of Sussex, Falmer, Brighton BN1 9GQ, UK; 4 Department of Biology, University of Leicester, University Road, Leicester LE1 7RH, UK

## Abstract

In desert locusts, increased population densities drive phenotypic transformation from the solitarious to the gregarious phase within a generation [1–4]. Here we show that when presented with odor-food associations, the two extreme phases differ in aversive but not appetitive associative learning, with solitarious locusts showing a conditioned aversion more quickly than gregarious locusts. The acquisition of new learned aversions was blocked entirely in acutely crowded solitarious (transiens) locusts, whereas appetitive learning and prior learned associations were unaffected. These differences in aversive learning support phase-specific feeding strategies. Associative training with hyoscyamine, a plant alkaloid found in the locusts’ habitat [5, 6], elicits a phase-dependent odor preference: solitarious locusts avoid an odor associated with hyoscyamine, whereas gregarious locusts do not. Remarkably, when solitarious locusts are crowded and then reconditioned with the odor-hyoscyamine pairing as transiens, the specific blockade of aversive acquisition enables them to override their prior aversive memory with an appetitive one. Under fierce food competition, as occurs during crowding in the field, this provides a neuroecological mechanism enabling locusts to reassign an appetitive value to an odor that they learned previously to avoid.

## Highlights

• Associative aversive learning is phase dependent, whereas appetitive learning is not
• Gregarization blocks the formation of new aversive memories
• Retention of previously acquired associative memories is unaffected by gregarization
• A behavioral feedback loop promotes override of a previously acquired aversive memory

## Results

Polyphenisms are an extreme form of phenotypic plasticity particularly common in insects, wherein a single genome has the capacity to produce distinct phenotypes to cope in different environments [7, 8]. Although extensive differences in morphology, physiology, and behavior have been characterized in species with alternative adult phenotypes, to our knowledge no studies have assessed whether they differ in their learning and memory capabilities. Variation in learning and memory capabilities among individuals [9–12], populations [13–16], and species [17–24] suggests that these capabilities are adapted to ecology and life history. Alternative adult phenotypes may likewise show differences in learning and memory capabilities that match their respective ecologies and life histories. This poses a problem, however, for species in which adults are capable of transforming from one phenotype to another: memories that are adaptive for the ecology and life history of one phenotype may not be so for the other.

Desert locusts (*Schistocerca gregaria*) can transform between two extreme phases, solitarious and gregarious, depending upon their local population density [1–4]. These phases show profound phenotypic differences and have distinct ecological demands [4]. Gregarization, the transformation of the solitarious into the gregarious phase, occurs over many timescales; some characters change rapidly [2, 3, 25], while others change slowly through epigenetic accumulation across generations [26]. Crucially, behavioral characters are the first to be modified: crowded solitarious locusts, referred to as “transiens” to indicate that they have begun to gregarize [1, 27], acquire most of the behavioral characteristics of the gregarious phase within just 4 hr of crowding [25]. These behavioral modifications include changes in feeding behavior that support a shift in the locusts’ antipredator strategy from crypsis to conspicuousness: whereas solitarious locusts reject toxic food, transiens and gregarious locusts readily feed on toxic plants to acquire and maintain unpalatability to vertebrate predators [28–30].

How do these density-dependent shifts in the locusts’ life history, which produce alternative phenotypes so distinct as to have been classified as distinct species until 1921 [31, 32], affect learning and memory? We addressed this question using associative learning paradigms that we have recently established in gregarious locusts [33, 34]. Using these paradigms, we determine whether the two extreme phases differ in their capability for learning and memory, and whether gregarization affects previously acquired memories and the acquisition of new ones.

We assessed odor preferences by giving each locust a single choice between vanilla odor and lemon odor in a Y maze [33] (see Figure S1 available online). In this paradigm, all locusts had comparable naive odor preferences irrespective of their phase state (G test with two degrees of freedom, G_2_ = 0.29; p = 0.865); about 70% of naive locusts selected the Y maze arm containing vanilla. This proportion was significantly different from the 50:50 distribution expected if there were no preference for either odor (G_1_ = 21.05; p < 0.001). Therefore, we designed the associative training to work against this naive preference for vanilla over lemon odor (see Supplemental Experimental Procedures).

Gregarious locusts tend to walk faster than their solitarious counterparts [2, 25]. Therefore, to verify the behavioral phase state of our experimental animals in subsequent experiments, we recorded the time that each locust took to reach the end of an arm in the Y maze. This latency was similar in gregarious and transiens locusts, and much shorter than in solitarious locusts because gregarized locusts walk faster (see Figure S2). The similar latencies in transiens and long-term gregarious locusts in the Y maze indicated that in all cases, crowding had induced full behavioral gregarization (Figures S2–S4).

### Aversive Learning

**Figure 1:**
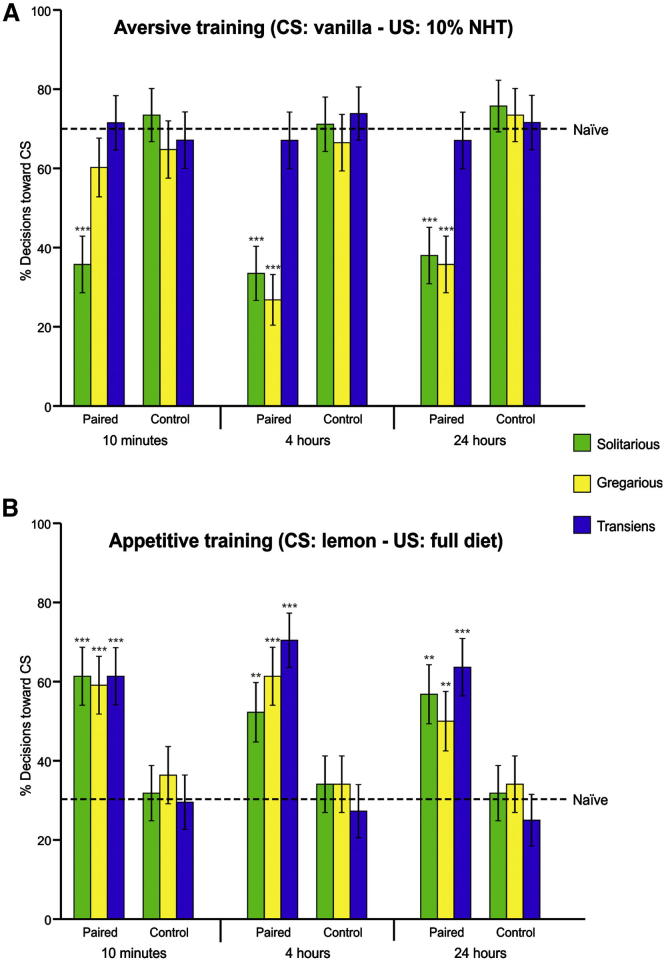
The Acquisition of Aversive, but Not Appetitive, Associative Odor Preferences in Desert Locusts Is Phase Dependent (A) A single aversive associative trial with vanilla odor as the conditioned stimulus (CS) and blank artificial diet containing 10% nicotine hydrogen tartrate (NHT) as the unconditioned stimulus (US) caused a phase-dependent change in the odor preference. As compared with the naive preference (dashed line), solitarious locusts showed strong odor aversion toward the CS as soon as 10 min after training (10 min, 34%, G_1_ = 23.61; 4 hr, 32%, G_1_ = 26.7; 24 hr, 36%, G_1_ = 20.73; all n = 44, p < 0.001), whereas in gregarious locusts, the aversive response was delayed (10 min, 59%, G_1_ = 2.22, p = 0.136; 4 hr, 25%, G_1_ = 37.26, p < 0.001; 24 hr, 33%, G_1_ = 23.61, p < 0.001). Transiens locusts did not show an aversive odor preference at any of the tested times when compared with the naive preference (10 min, 30%, G_1_ = 0.01; 4 hr, 34%, G_1_ = 0.29; 24 hr, 34%, G_1_ = 0.29; all n = 44, p > 0.589). When locusts of all three phases were trained with only the CS, their choices were indistinguishable from naive preference (solitarious: 10 min, 27%; 4 hr, 30%; 24 hr, 25%; gregarious: 10 min, 36%; 4 hr, 34%; 24 hr, 27%; transiens: 10 min, 34%; 4 hr, 27%; 24 hr, 30%; all n = 44, G_1_ < 0.74, p > 0.390). (B) Four appetitive associative trials with lemon odor as the CS and artificial diet as the US caused a significant increase in the preference for the CS, regardless of the locusts’ phase state, compared with that of naive locusts (solitarious: 10 min, 61%; 4 hr, 52%; 24 hr, 57%; gregarious: 10 min, 59%; 4 hr, 61%; 24 hr, 50%; transiens: 10 min, 61%; 4 hr, 71%; 24 hr, 64%; all n = 44, G_1_ > 9.18, p < 0.006). The odor preference of locusts trained with CS only was no different than expected from the naive preference (solitarious: 10 min, 32% lemon over vanilla; 4 hr, 34%; 24 hr, 32%; gregarious: 10 min, 36%; 4 hr, 34%; 24 hr, 34%; transiens: 10 min, 30%; 4 hr, 27%; 24 hr, 25%; all n = 44, G_1_ < 0.74, p > 0.39). Error bars represent ±SE. ^∗∗^p < 0.01; ^∗∗∗^p < 0.001.

Does a locust’s phase affect its ability to learn an association between an odor and toxic food? Locusts were trained with a single presentation of vanilla odor (conditioned stimulus, CS) paired with artificial nonnutritious diet (blank diet) containing nicotine hydrogen tartrate (NHT; unconditioned stimulus, US). A training trial involved 5 s of CS presentation followed by 20 s of simultaneous CS/US presentation. After training, locusts were returned to their original cages to await testing (see Supplemental Experimental Procedures), when they were given the choice between the CS (vanilla) and a novel stimulus (NS, lemon odor) in the Y maze. Ten minutes after training, only 34% of solitarious locusts chose the CS, indicating a clear learned aversion compared with naive locusts (Figure 1A). In contrast, however, 59% of gregarious locusts still chose the CS, indistinguishable from their naive preference (Figure 1A). A direct comparison confirmed that solitarious locusts chose the CS over the NS significantly less often than did their gregarious counterparts (heterogeneity G value with one degree of freedom, G_H1_ = 5.60; p = 0.02). Four hours and 24 hr after training, however, there was no difference between phases in the choices they made (4 hr, G_H1_ = 0.50; 24 hr, G_H1_ = 0.05; both n = 44, p > 0.478); irrespective of their phase, approximately two-thirds of locusts now chose the NS over the CS, twice as many as expected from the naive preference (Figure 1A). When locusts were trained with the CS in the absence of the US, their choices were indistinguishable from naive locusts at all retention times, irrespective of their phase (CS-only control; Figure 1A). Thus, both phases learn to aversively associate dietary NHT with an odor following a single paired trial and do not differ in how long they retain this memory. However, the two phases differ in when they first express this aversion; solitarious locusts show a conditioned aversion 10 min after training, whereas their gregarious counterparts do not. Such a difference is probably related to a phase-specific difference in the acquisition mechanism [34].

We then repeated this experiment with solitarious locusts that had been crowded for 24 hr prior to training. Remarkably, these transiens locusts showed no sign of increased aversion to the CS at any time point tested; only about one-third decided against the CS, a rate similar to the naive preference (Figure 1A) and significantly lower than that of their solitarious (10 min, G_H1_ = 11.94; 4 hr, G_H1_ = 10.44; 24 hr, G_H1_ = 7.80; all p < 0.005) and gregarious counterparts (4 hr, G_H1_ = 15.31; 24 hr, G_H1_ = 9.07; all p < 0.003). The odor choices of transiens locusts trained with CS only were likewise indistinguishable from naive choices at all retention times (CS-only control; Figure 1A). Thus, gregarization impairs the manifestation of the conditioned aversion. However, this experiment does not resolve whether this block is at the level of memory acquisition, retention, or retrieval.

### Appetitive Learning

To investigate whether phase differences are restricted to aversive learning, we trained solitarious, gregarious, and transiens locusts with four trials in which lemon odor as CS was paired with artificial full diet as US (see Supplemental Experimental Procedures). We used four CS/US trails because gregarious locusts do not retain the memory induced by single-trial appetitive training for 24 hr [33]. Ten minutes, 4 hr, or 24 hr after training, the locusts were given the choice between CS and vanilla as the NS to determine whether the memory had been retained. At each retention time, about 60% chose the CS over the NS, significantly more than expected from the naive preference (Figure 1B). The choices were similar among all three groups of locusts at each retention time (10 min, G_2_ = 0.06; 4 hr, G_2_ = 3.09; 24 hr, G_2_ = 1.67; all p > 0.213), indicating that phase affected neither the acquisition nor the retention of this appetitive memory. The choices of locusts trained with CS only were no different than expected from the naive preference and were similar across the three phase states (10 min, G_2_ = 0.48; 4 hr, G_2_ = 0.64; 24 hr, G_2_ = 0.95; all p > 0.623) (CS-only control; Figure 1B). Thus, all phases show a comparable memory of the appetitive associations between an odor and a food reward. Moreover, acute crowding of solitarious locusts to gregarize them does not inhibit appetitive associative learning, demonstrating that the impairment of aversive associative learning is specific rather than a general impairment of learning.

### Acquired Memories Are Robust to Gregarization

**Figure 2:**
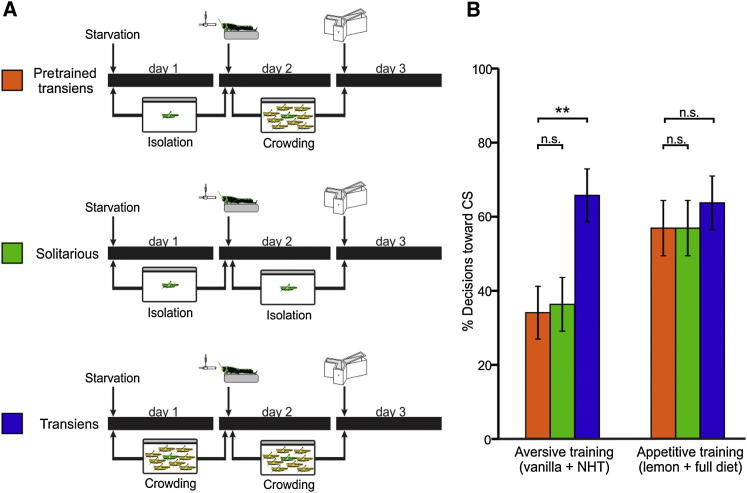
Associative Memories Acquired by Solitarious Locusts Are Not Disrupted by Gregarization (A) Training and testing protocols used to test the persistence of associative memories throughout gregarization. The three groups of locusts were either appetitively or aversively trained. (B) Appetitive conditioning caused an increase in the preference for the CS in pretrained transiens locusts similar to that made by solitarious and transiens locusts (G_1_ = 0 and G_1_ = 0.43, respectively; both p > 0.513; α′ = 0.025). Aversively conditioned pretrained transiens locusts avoid the CS, showing a conditioned response similar to that of solitarious locusts (G_1_ = 0.05; p = 0.823; α′ = 0.025) but higher than that of transiens locusts (G_1_ = 9.07; p = 0.003; α′ = 0.025). Error bars represent ±SE. ^∗∗^p < 0.01; n.s., not significant.

Differences in the behavior and life history of solitarious and gregarious locusts mean that memories acquired by solitary locusts may cease to be useful or may even be deleterious if they are maintained during and after gregarization. Indeed, crowding solitary locusts causes substantial and rapid changes in their neurochemistry [35], which could disrupt memories acquired previously. To assess the effects of crowding, we trained solitarious locusts either appetitively or aversively exactly as described above and then crowded them immediately for 24 hr. Hereafter we refer to these locusts as “pretrained transiens.” We compared the choices of these pretrained transiens locusts after 24 hr with those of solitarious locusts maintained in uncrowded conditions after training, and transiens locusts that were trained *after* 24 hr of crowding (Figure 2A). After appetitive conditioning, the majority (57%) of the pretrained transiens locusts chose the CS over the NS, similar to the percentage of choices made by solitarious (57%) and transiens (64%) locusts with equal training (Figure 2B). After aversive training, only 34% of the pretrained transiens locusts chose the CS over the NS, a similar percentage to that of aversively trained solitarious locusts (36%) and significantly lower than that of transiens locusts (66%) (Figure 2B). Thus, both appetitive and aversive associative memories acquired by solitarious locusts are retained during gregarization and can be retrieved afterward. Consequently, these results also indicate that the absence of aversion in transiens locusts trained after crowding is due to a temporary suppression of memory acquisition that occurs during gregarization and affects aversive acquisition specifically.

### Phase-Dependent Reinforcement Value of Hyoscyamine

**Figure 3:**
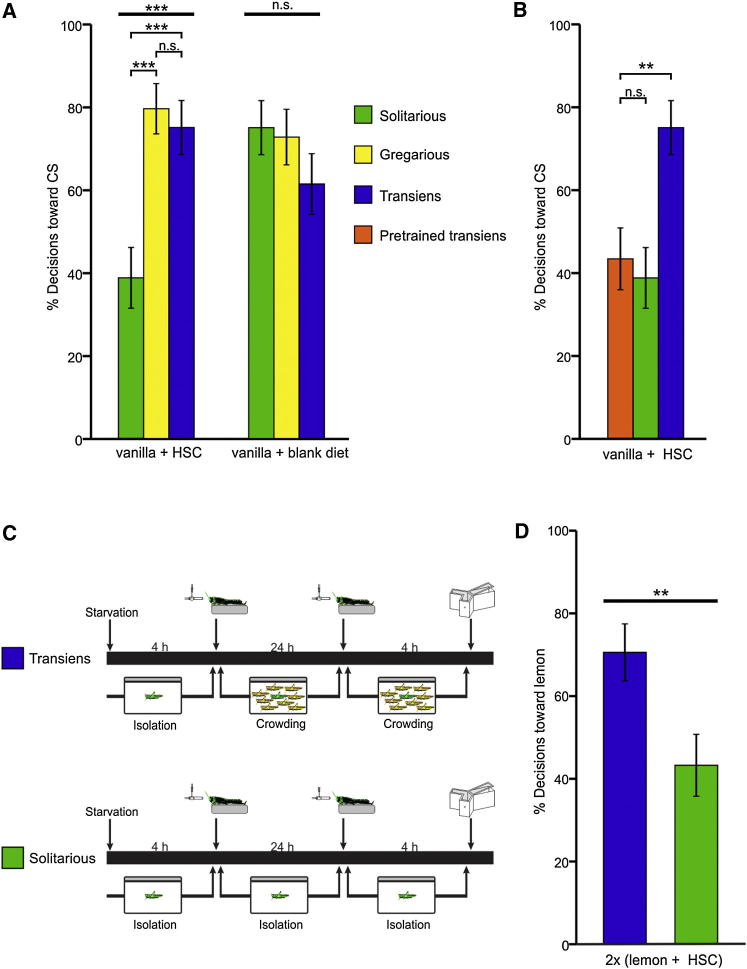
Associative Training with Hyoscyamine Elicits a Phase-Dependent Odor Preference in Desert Locusts Unlike transiens and gregarious locusts, solitarious locusts acquire an aversive hyoscyamine (HSC) memory that is retained after gregarization. A single further training trial after crowding, however, is sufficient for the now-transiens locusts to switch the value of the HSC-associated odor from aversive to appetitive. (A) A single associative training trial with vanilla odor as the CS and blank artificial diet containing 2% HSC as the US caused a phase-dependent change in the odor preference in a 4 hr retention test (G_2_ = 19.12, n = 44 each; p < 0.001), whereas training with blank diet did not (G_2_ = 2.19, n = 44 each; p = 0.335). (B) The associative memory acquired by pretrained transiens locusts was not altered after gregarization; these locusts showed a conditioned preference similar to that of solitarious locusts (G_1_ = 0.19; p = 0.664; α′ = 0.025) but lower than that of transiens locusts (G_1_ = 9.41; p = 0.002; α′ = 0.025). (C) Training and testing protocol used to test the gregarization-dependent reinforcement value of HSC. (D) After double-training with two identical CS/HSC pairings, the percentage choosing the CS was significantly greater in transiens locusts than in than solitarious locusts (G_1_ = 6.763; p < 0.01). Error bars represent ±SE. ^∗∗^p < 0.01; ^∗∗∗^p < 0.001; n.s., not significant.

Solitarious locusts avoid food containing hyoscyamine (HSC), a toxic alkaloid found in plants native to their habitat [5, 6], whereas recently gregarized transiens locusts preferentially ingest food containing HSC [29, 30]. To test whether the reinforcement value of HSC in associative learning reflects this phase-dependent feeding preference, we trained solitarious, gregarious, and transiens locusts with a single CS/US trial with vanilla odor paired with 2% HSC in blank diet. Locusts trained with a CS/US trial in which the US was blank diet lacking HSC served as controls. Locusts were tested 4 hr later, when both appetitive and aversive memories can be observed, by making them choose between the CS and lemon as NS (see Supplemental Experimental Procedures). Pairing the CS with HSC as US elicited different olfactory responses in the three phases, whereas pairing with blank diet did not (Figure 3A). Among the solitarious locusts, only a minority (39%) chose the CS over the NS after training with HSC as US, compared with a majority (75%) after training with blank diet as US (G_1_ = 12.16; p < 0.001). The HSC-trained solitarious locusts also avoided the CS more often than their gregarious and transiens counterparts (G_1_ = 15.78 and G_1_ = 12.16, respectively; both p < 0.001; α′ = 0.017) (Figure 3A). The percentages of HSC-trained gregarious (79%) and transiens (75%) locusts that chose the CS were not significantly different from one another (G_1_ = 0.26; p = 0.611; α′ = 0.017) or from the CS preference of the gregarious (73%) and transiens (61%) locusts trained with blank diet (G_1_ = 0.56 and G_1_ = 1.90, respectively; both p > 0.170) (Figure 3A). This indicates that solitarious locusts experience HSC as a negative reinforcer and consequently form an aversive association, whereas gregarious and transiens locusts experience HSC as a neutral or appetitive stimulus.

This phase-dependent valuation of HSC would seem to pose a problem for solitarious locusts that learn to associate an odor with food containing HSC but undergo gregarization subsequently. These locusts need to seek out and ingest food containing HSC to protect themselves from predators, but they will retain their aversive memories during gregarization. To determine how gregarized locusts switch the value of the association, we trained solitarious locusts with a single CS/US trial of vanilla odor paired with 2% HSC and crowded them for 24 hr prior to testing (pretrained transiens). We compared their choices with those of solitarious and transiens locusts trained in the same way (Figure 2A). Of these pretrained transiens locusts, ∼43% chose the CS, a percentage similar to that of solitarious locusts but significantly lower than that of transiens locusts (Figure 3B). This confirms that associations acquired prior to gregarization are maintained. Consequently, newly gregarized locusts retain aversive associations that are no longer appropriate for their new ecological circumstances.

What mechanism enables these locusts to start to ingest the toxins they need to make them distasteful to predators? Our results with NHT (Figures 1A and 2B) and HSC (Figure 3A) showed that crowding impairs the acquisition of aversive associations. Consequently, for transiens locusts that have already formed an aversive association between HSC and an odor, HSC may no longer act as a negative reinforcer upon subsequent exposure. To test this hypothesis, we starved solitarious locusts for 4 hr prior to a single CS/US training trial of lemon odor paired with 2% HSC. One half of these locusts were then crowded for 24 hr, while the other half were returned to their isolated cages for the same period of time. Both the transiens and solitarious locusts were then trained for a second time with the same CS/US pairing (lemon/HSC), and their odor choice was tested 4 hr later (Figure 3C). Of these double-trained transiens locusts, ∼71% chose the CS, significantly more than the 43% of uncrowded solitarious locusts (Figure 3D). Thus, the double-trained transiens locusts no longer show an aversion to the CS. This demonstrates that, despite retaining an aversive association between an odor and HSC during gregarization, transiens locusts can update this association upon subsequent reexposure to the same odor paired with food containing HSC. Therefore, the experience of crowding alone transforms a further exposure to the odor-toxin pairing, which to solitarious locusts is a second aversive training trial, into an appetitive training trial that overrides their previously formed aversive association. This update of the memory that has been formed prior to gregarization is enabled by the specific blockade of aversive memory acquisition that characterizes the period of transition to gregariousness.

## Discussion

The profound differences in the acquisition of aversive odor-food associations between phases can be interpreted as adaptive in their different ecological niches. Being able to rapidly form aversive associations, rather than waiting to determine the consequences of ingestion, should help solitarious locusts maintain their narrow dietary preferences and avoid ingesting toxins [29, 30]. This rapid acquisition of aversive associations in solitarious locusts is probably taste-mediated, because memories acquired by postingestive feedback take longer to manifest [34]. Conversely, the absence of this rapid, taste-mediated aversive learning mechanism in gregarious locusts matches their broader diet and their active ingestion of toxic plants to acquire and maintain unpalatability [28–30]. The delayed aversion shown by gregarious locusts is mediated by a postingestive mechanism operating independently of gustation [34, 36, 37]. That gregarious locusts form postingestive aversive memories to NHT-associated odors at all suggests that they do so to strike a balance between the benefits (nutritional and defensive) gained from ingesting toxic plants and the putative cost of the toxic malaise they incur.

Recently gregarized transiens locusts lack both rapid taste-mediated and long-latency postingestive aversive learning. The suppression of the latter may indicate that their malaise tolerance is elevated greatly, permitting them to ingest greater amounts of toxins despite the cost without forming aversive associations. However, transiens locusts can still form appetitive associations, demonstrating that the blockade affects the acquisition of aversive associations specifically. Furthermore, they can still recall both aversive and appetitive associations. This implies that the neuronal circuits responsible for consolidating and maintaining associative memories survive the extensive neurochemical modifications that accompany gregarization [35]. There are precedents for socially and toxin-induced selective memory blockades in other insects [38–41]. Unlike in previous examples, however, in desert locusts the blockade accompanies a profound shift in life history and is brought about solely by the presence of conspecifics.

Given the current understanding of the circuits that are the substrate of olfactory associations in the insect brain (for reviews, see [42, 43]), it is unsurprising that transiens locusts are unable to switch a specific memory from being aversive to appetitive. This inability is exposed in a laboratory setting where the locusts are asked to perform the switch in vacuo. We show here how simple learning mechanisms combined with hunger and competition for food allow transiens locusts to override previously acquired aversive associations when reexposed to the same association. This override involves a behavioral feedback loop in which the transiens locusts effectively retrain themselves: being hungry, they ingest food that contains the toxin. In doing so, they can no longer reinforce their existing aversive memory to the toxin, but they can form an appetitive memory to the food and/or water that accompanies the toxin. Consequently, they form an appetitive association with the odor that they had previously associated with an aversive toxin.

What is the likely ecological significance of the suppression of aversive learning during the transition to gregariousness? An increase in local population density produces intense food competition, forcing solitarious locusts together and thereby triggering their gregarization. The resulting transiens locusts continue to compete fiercely for dwindling food resources, ingesting all available plants [6, 27, 44]. However, they preferentially ingest plants with toxic compounds to become unpalatable to predators [28–30]. Thus, the change in life history from solitarious to gregarious entails a change in the ecological value of toxic plants. Yet locusts retain associations throughout this change in life history, including aversive memories to toxin-containing food that are no longer ecologically appropriate. When situated in their behavioral environment, a relearning mechanism that comprises the selective blockade of aversive memory formation coupled with hunger and competition for food could enable transiens locusts to assign an appetitive value to an odor they previously learned to avoid. Thus, under the conditions that drive gregarization in the field, the specific blockade of aversive acquisition enables locusts to update their memories to match the new ecological value of toxic food plants.

### Conclusion

This is the first demonstration of differences in learning capability between alternative adult phenotypes, and also of transient modifications in learning capability during the process of phenotypic transformation; the latter modifications go beyond the differences observed between the two extreme phenotypes. When presented with odor-food associations, long-term solitarious and gregarious desert locusts show comparable memory retention, but they differ in aversive memory acquisition, with solitarious locusts manifesting aversion sooner than gregarious locusts. This difference in aversive learning between the two phases may support their distinct feeding ecologies, helping solitarious locusts to avoid ingesting toxic compounds while allowing gregarious locusts to maintain their chemical defense by ingesting toxins without forming aversions. Yet this profound and seemingly highly adaptive difference in learning capability has its simple mechanistic basis in the selective suppression of taste-mediated, but not postingestive, learning in the gregarious phase. The specific and rapid changes in learning capability that occur temporarily during the process of phenotypic transformation are similarly tailored to the specific requirements of the life-history strategy of the transitional phenotype. As with the long-term differences, these changes have their basis in modifications to simple learning rules. However, when embedded in the context of the field, these simple rule changes provide a neuroecological mechanism for something that the locust cannot perform in vacuo: namely, switching the learned value of an odor from aversive to appetitive.

## Supplemental Information

Document S1. Figures S1–S4 and Supplemental Experimental Procedures
