# Supplementary material for: Phenotypic Transformation Affects Associative Learning in the Desert Locust
Source: Curr Biol. 2013 Dec 2;23(23):2407–12. doi: 10.1016/j.cub.2013.10.016 (PMC4024192; doi:10.1016/j.cub.2013.10.016)
Supplement: Document S1. Figures S1–S4 and Supplemental Experimental Procedures [file mmc1.pdf]

Current Biology, Volume *23*

Supplemental Information

Phenotypic Transformation Affects Associative Learning in the Desert Locust

Patrício M.V. Simões, Jeremy E. Niven, and Swidbert R. Ott

Inventory of Supplemental Information

Figure S1. Diagram of the Y maze used in testing.

Figure S2. Test latencies of locusts trained with an olfactory associative conditioning. Related to Figure 1.

Figure S3. Test latencies of pre-trained transient locusts. Related to Figure 2.

Figure S4. Test latencies of locusts trained with HSC. Related to Figure 3.

Supplemental Experimental Procedures. Here we present the detailed experimental procedures for training and testing the desert locusts, the method used to measure the locusts' test latencies, and the statistical analysis of the results.

Supplemental References

Figure S1.

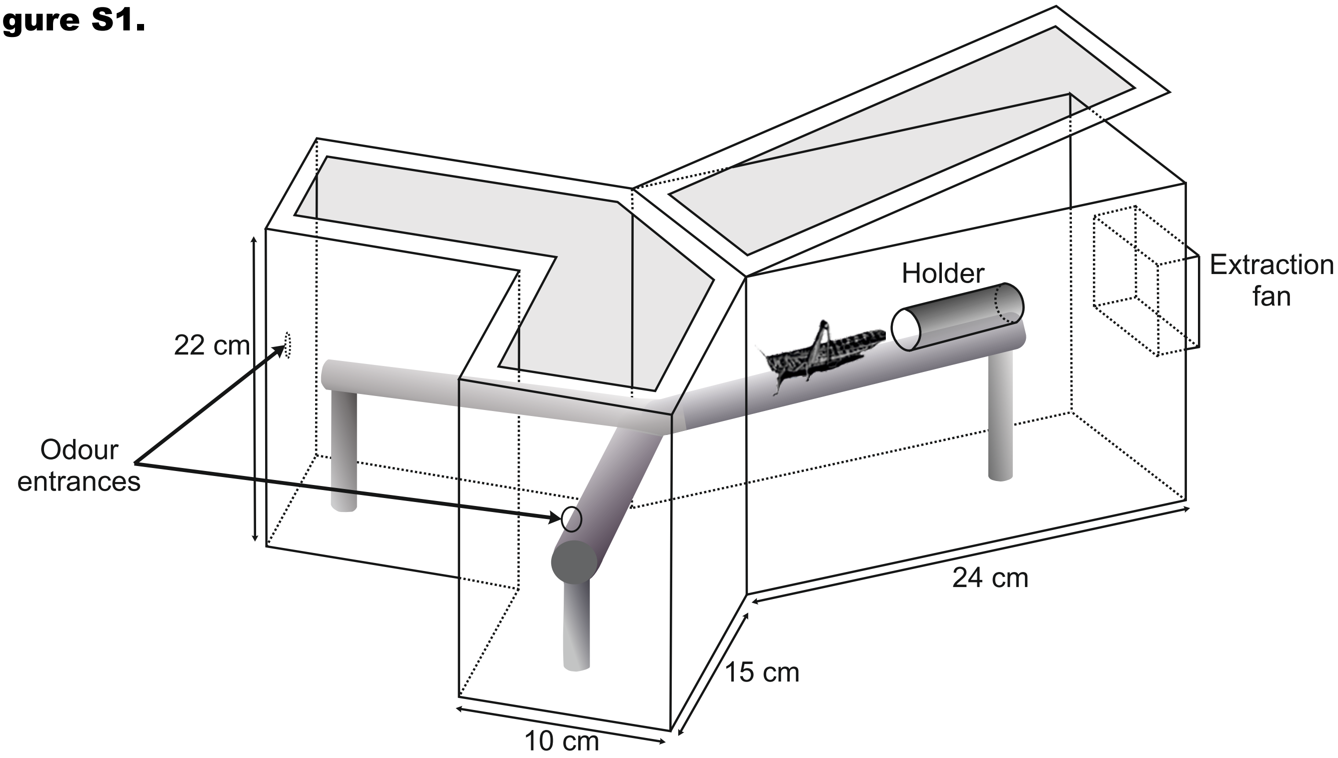

Figure S2.

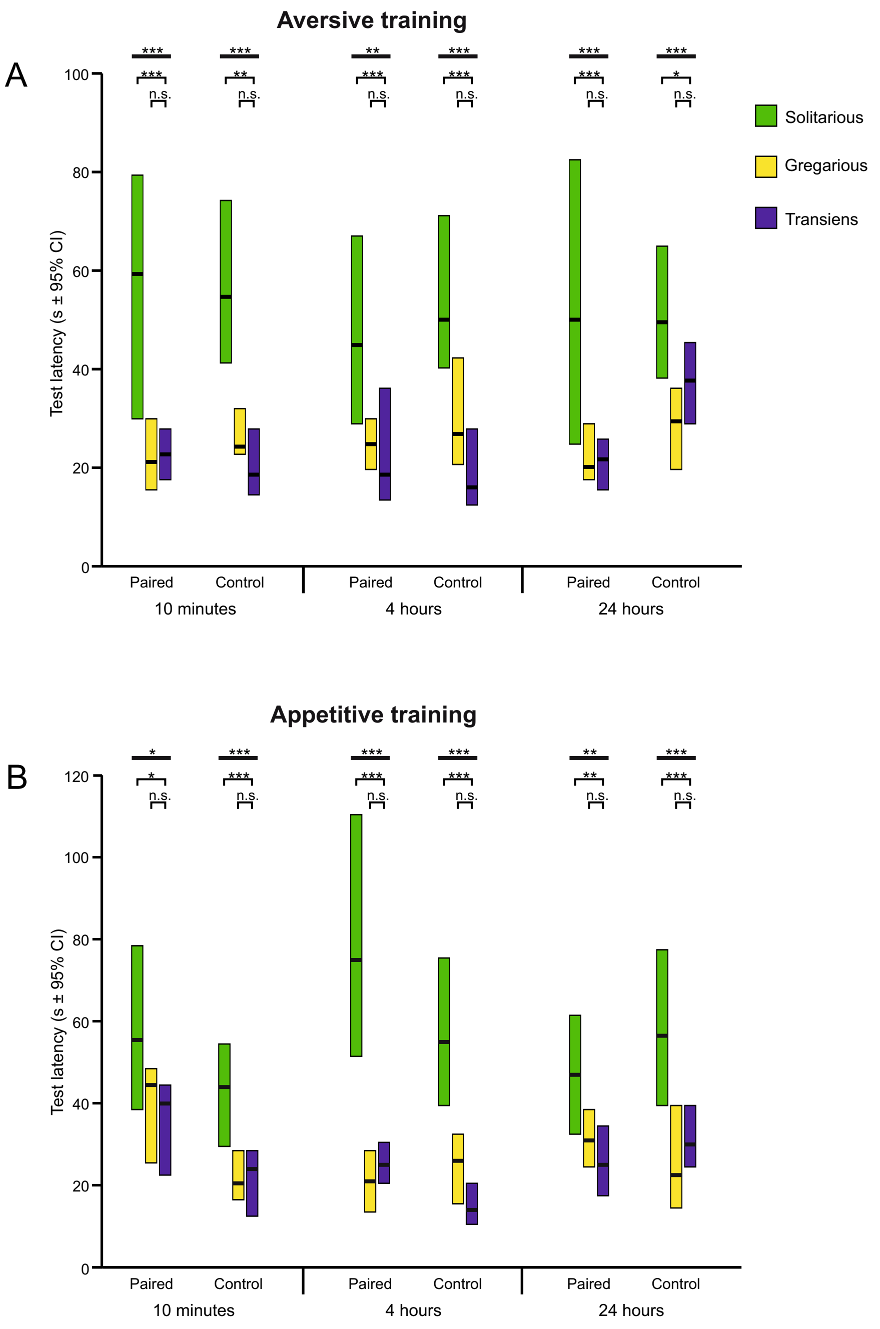

Aversive training
A
Test latency (s ± 95% CI)
100
80
60
40
20
0
*** *** n.s.
*** ** n.s.
** *** n.s.
*** *** n.s.
*** *** n.s.
*** * n.s.
Paired
Control
10 minutes
4 hours
24 hours
Solitarious
Gregarious
Transiens
Appetitive training
B
120
100
* * n.s.
*** *** n.s.
** ** n.s.

Figure S3.

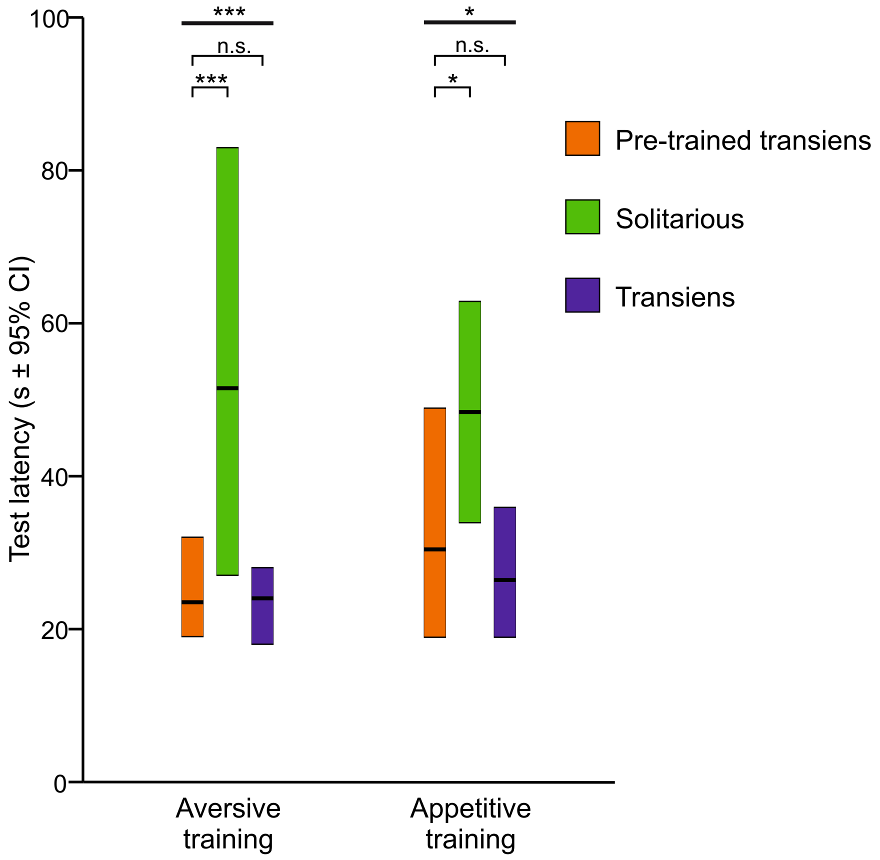

***
n.s.
***
*
n.s.
*
100
80
60
40
20
0
Test latency (s ± 95% CI)
Pre-trained transiens
Solitarious
Transiens
Aversive training
Appetitive training

Figure S4.

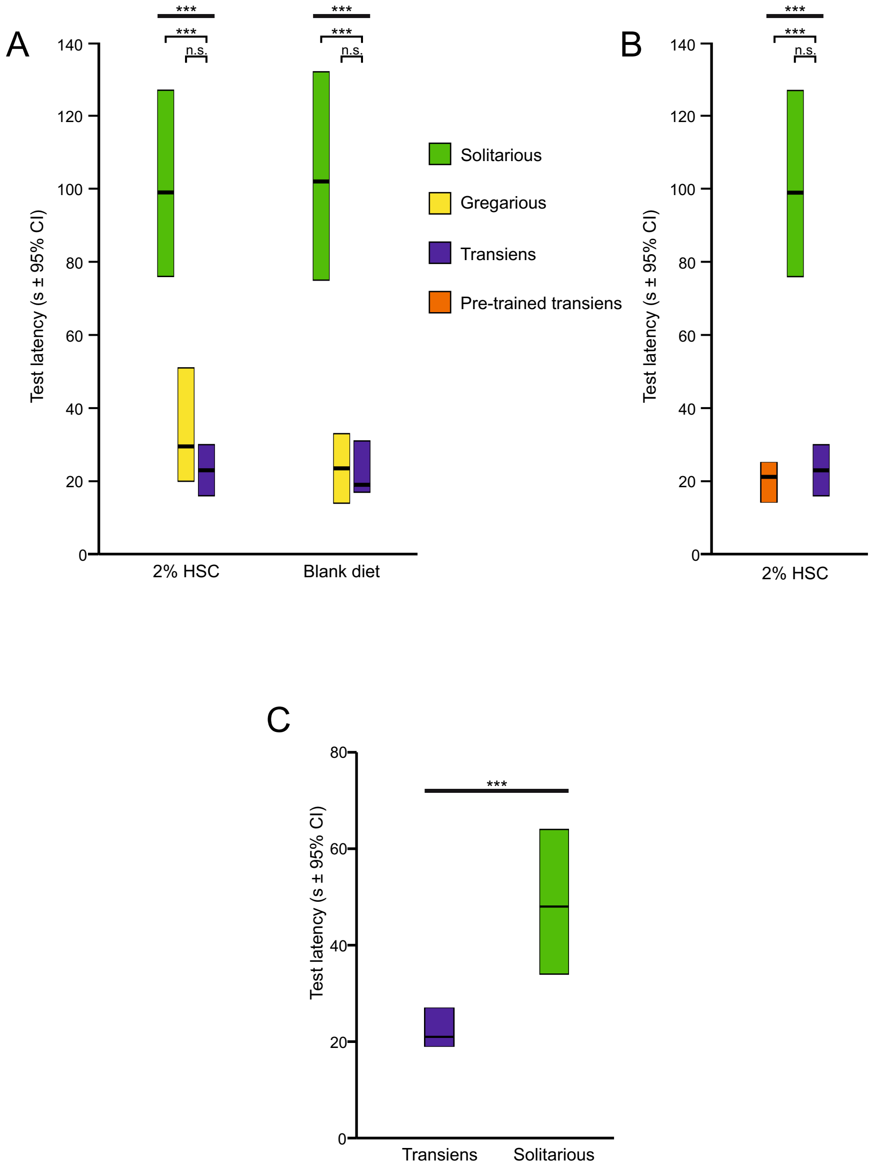

A
B
C
***
n.s.
Test latency (s ± 95% CI)
0
20
40
60
80
100
120
140
2% HSC
Blank diet
Solitarious
Gregarious
Transiens
Pre-trained transiens
Transiens
Solitarious

Supplemental Data

Figure Legends

Figure S1. Diagram of the Y-maze as used for testing odour preferences throughout this study.

Figure S2. Test latencies of locusts after olfactory associative conditioning depend on their crowding history. Test latencies were measured as the time each locust took from exiting the holder until reaching the end of either arm in the Y-maze. In all tests, solitarious locusts crowded for 24 h (transiens) walked faster in the Y-maze than solitarious and similarly to gregarious locusts.

(A) Test latencies after training with CS paired with 10% NHT as US (CS/US) or with CS only (controls). The latencies of transiens locusts were similar to those of gregarious locusts (Figure S2A; CS/US – 10 min: *U*= 942.5; 4h: *U*=872.5; 24 h: *U*=956; CS only – 10 min: *U*=838.5; 4 h: *U*=772; 24 h: *U*=803; all P>0.102; α'=0.025) and shorter than those of solitarious locusts (Figure S2A; CS/US – 10 min: Mann-Whitney *U*-test, *U*= 442, P<0.001; 4h: *U*=618, P=0,003; 24 h: *U*=535, P<0.001; CS only – 10 min: *U*=349, P<0.001; 4 h: *U*= 384.5, P<0.001; 24 h: *U*=692.5, P=0,021; Dunn–Sidak correction, α'=0.025). Moreover, the test latencies were independent of which odour the locusts chose, irrespective of whether they had been trained with CS/US (solitarious – 10 min: *U*= 169; 4h: *U*=193; 24 h: *U*=185; gregarious – 10 min: *U*=159; 4 h: *U*=127; 24 h: *U*=184.5; transiens – 10 min: *U*=182.5; 4 h: *U*=169 ; 24 h: *U*=188.5; all P>0.073) or with CS only (solitarious – 10 min: *U*=146.5 ; 4h: *U*=161; 24 h: *U*=165; gregarious – 10 min: *U*=151.5; 4 h: *U*=187.5 ; 24 h: *U*=190; transiens – 10 min: *U*=209; 4 h: *U*=167; 24 h: *U*=201; all P>0.076).

(B) Test latencies after training with CS paired with full diet as US (CS/US) or with CS only (controls). In all tests, the latencies of transiens locusts were similar to those of gregarious locusts (Figure S2B; CS/US – 35 10 min: *U*=891.5; 4h: *U*=902.5; 24 h: *U*=809.5; CS only – 10 min: *U*=901.5; 4 h: *U*=774.5; 24 h: *U*=859; all P>0.106; α'=0.025) and shorter than those of solitarious locusts (Figure S2B; CS/US – 10 min: *U*= 674, P=0.014; 4h: *U*=324, P<0.001; 24 h: *U*=644, P=0.007; CS only – 10 min: *U*=514, P<0.001; 4 h: *U*= 407, P<0.001; 24 h: *U*=540, P<0.001; α'=0.025). The test latencies were independent of which odour the locusts chose, irrespective of whether they had been trained with CS/US (solitarious – 10 min: *U*=216 ; 4h: *U*=193; 24 h:

U=217.5; gregarious – 10 min: U=177; 4 h: U=181.5; 24 h: U=184; transiens – 10 min: U=214.5; 4 h: U=174; 24 h: U=211; all P>0.173) or with CS only (solitarious – 10 min: U=192 ; 4h: U=188; 24 h: U=164.5; gregarious – 10 min: U=204.5; 4 h: U=212 ; 24 h: U=217; transiens – 10 min: U=157; 4 h: U=183.5; 24 h: U=133; all P>0.196).

Dispersion bars represent a 95% Confidence Interval of the median. * P<0.05; ** P<0.01; *** P<0.001.

Figure S3. Test latencies of appetitively or aversively trained locusts were significantly different across the three groups of locusts that we compared: solitarious, transiens and pre-trained transiens (appetitive: $K2$=8.79, P=0.012; aversive: $K2$=17.53, P<0.001). Pre-trained transiens locusts walked faster than solitarious (appetitive: U=683.5, P=0.018; aversive: U=534, P<0.001; α'=0.025) and as fast as transiens locusts (appetitive: U=915.5, P=0.673; aversive: U=952.5, P=0.897; α'=0.025).

Dispersion bars represent a 95% Confidence Interval of the median. * P<0.05; *** P<0.001.

Figure S4. Test latencies of locusts trained with HSC. All transiens groups walked faster in the Y-maze than the solitarious locusts.

(A) Test latencies after training with HSC-containing or blank diet. The different phases showed significant differences in the test latencies, irrespective of whether they were trained with HSC or blank diet (HSC: $K2$=41.55; blank diet: $K2$=42.79, both P<0.001). Transiens locusts walked faster than solitarious locusts (HSC: U=228; blank diet: U=291.5; both P<0.001; α'=0.025), and as fast as gregarious locusts (HSC: U=775.5, P=0.112; blank diet: U=920, P=0.688; α'=0.025).

(B) The test latencies of the pre-trained transiens locusts were shorter than those of the solitarious locusts (U=185, P<0.001; α'=0.025) but similar to those of the transiens locusts (U=897.5, P=0.556; α'=0.025).

(C) The test latencies of the double-conditioned transiens locusts were shorter than those of solitarious locusts (U=439; P<0.001).

Supplemental Experimental Procedures

Animals

We used adult desert locusts (*Schistocerca gregaria* Forskål, 1775) of either sex, bred at the Department of Zoology, University of Cambridge, UK. Long term solitarious and gregarious locusts were maintained on an identical diet of fresh seedling wheat and dry wheat germ under a 12:12 h photoperiod. Gregarious locusts were taken from a colony maintained under crowded conditions for many generations. Batches of approximately 40 gregarious locusts were transferred to a heated holding tank (24 x 30 x 20 cm, 35°C) and starved for 24 h. They remained in the holding tank to maintain crowding except during training and testing. Solitarious locusts were produced from the gregarious stock by rearing them in individual cages under visual, tactile and olfactory isolation from conspecifics for two to four generations [S1]. Prior to training, solitarious locusts were starved for 24 h in their individual rearing cages. After training, solitarious locusts were returned to their individual cages until testing. The effect of gregarization was tested by crowding the locusts after training. Transiens locusts were produced by crowding long-term solitarious locusts in a heated holding tank with about 40 gregarious adults for 24 h. Physical interaction between all locusts was unrestrained. No food was given during this period. After training, transiens locusts were returned to the crowded holding tank to prevent re-solitarization.

Aversive Olfactory Associative Training with Nicotine

Locusts were secured in modelling clay and allowed to rest for 5 minutes prior to training. The Conditioned Stimulus (CS) for aversive training was vanilla extract (Dr Oetker, Thorpe Park, Leeds, UK). The CS (25 μl) was placed on 1 cm^2 of filter paper inside a plastic tube connected to an air pump. The end of the tubing was directed towards the locusts' antennae and placed approximately 5 cm away.

The aversive Unconditioned Stimulus (US) was a blank artificial diet containing 10% nicotine hydrogen tartrate (NHT; Sigma-Aldrich; CAS 65-31-6). The blank diet was obtained by replacing the nutrients of the standard locust artificial diet with the same weight of cellulose, which is indigestible to locusts [S2]. The US was mixed with an equal weight of water and delivered to the locust's mouth on a small metal spatula.

A training trial consisted of a 5 s CS presentation followed by a 20 s simultaneous CS/US presentation. A 20 s pre- and post-trial resting time was given. Animals that refused to feed in any of the trials were excluded. There were 44 locusts in each experimental group. After training, the locusts were removed from the modelling clay and returned to the holding tank or individual cage to await testing.

Appetitive Olfactory Associative Training

The procedure for olfactory appetitive associative training was identical to that for aversive associate training except for the stimuli to which the locusts we exposed. The CS for appetitive training was lemon extract (Holland & Barrett, Nuneaton, Warwickshire, UK), whilst the US was the standard locust artificial diet, with an equal protein to carbohydrate ratio [S3]. The inter-trial interval was 5 minutes.

Aversive Olfactory Associative Training with Hyoscyamine

The procedure for olfactory aversive associative training with hyoscyamine (HSC) was identical to that involving nicotine except that the US was 2% of HSC (MP Biomedicals; CAS 101-31-5) in blank diet. The percentage of HSC in the diet was based on previous studies [S4,S5].

Odour Preference Test

All locusts were submitted to a single odour preference test in a Y-shaped arena containing a raised wooden Y-shaped rod on which they had to walk. Vanilla and lemon odours were presented in each of the two decision arms. The arena, odour delivery set-up and test procedure are described elsewhere [S6]. Half of the animals were tested with vanilla odour in the right arm and the other half with vanilla in the left. A locust was placed inside a holding tube positioned on the stem of the Y bar and left undisturbed for a few seconds before the airflow inside the Y-maze was turned on. Each locust was allowed 5 minutes to exit the tube and make a choice. A choice was made when a locust walked to the end of an arm and touched the wall with either the front legs or the antennae. Locusts that failed to make a decision within 5 minutes, or that fell off or jumped from the rod were discarded. After each test, the locust was removed from the Y-maze and the wooden rod was wiped with 70% alcohol to disperse any pheromone cues and left to dry.

Test Latency

Behavioural gregarization in desert locusts includes rapid and striking modifications in locomotor behaviour as well as a change from being repelled by to being attracted to conspecifics [S7, S8]. To confirm that, in our experiments on transiens locusts, 24 hours of crowding had indeed induced behavioural gregarization in the previously solitarious locusts (i.e., that they were indeed transiens), we measured the time they took from exiting the holder until reaching the end of either arm in the Y-maze (test latency) and compared it with the latencies of the two extreme phases. The test latency is essentially a measure of locomotor activity within the Y-maze because it is unknown at which point within the maze individual locusts comparing odours and make a decision. Latencies were highly variable, as some locusts reached the end of the Y-maze within 10 seconds, whilst others of the same phase and equal training took more than 4 minutes.

Statistical Analysis

The locusts' odour preference in the Y-maze and the latency of the olfactory decision were recorded. In each experimental group both sex and the position of the odour relative to the decision arms were balanced. *G*-tests for independence were used to determine whether sex affected the locusts' behaviour. *G*-tests for goodness of fit were used to determine whether there was divergence from an expected 50% decision for each arm side. No significant sex differences or side biases were found in any of the experiments, consequently the data were pooled with respect to sex and odour position in the arms of the Y-maze.

The odour preference after training was compared against that of naïve locusts as an extrinsic null hypothesis in *G*-tests for goodness of fit, with the sources of heterogeneity within significant data sets being analysed using unplanned tests of homogeneity [S9]. All other odour preference comparisons between experimental groups were by *G*-tests for independence. The Dunn Sidak correction was applied to determine significance in multiple comparisons. Comparisons of the time taken by locusts to walk to the end of a Y-maze arm, or test latency, between experimental groups were made using non-parametric Mann-Whitney *U*-tests or Kruskal–Wallis tests because the data did not meet the assumptions of normality.

Supplemental References

S1. Roessingh, P., Simpson, S.J., and James, S. (1993). Analysis of phase-related changes in behavior of desert locust nymphs. Proc. R. Soc. B *252*, 43–49.

S2. Dadd, R.H. (1960). The nutritional requirements of locusts - I Development of synthetic diets and lipid requirements. J. Insect Physiol. *4*, 319-347.

S3. Simpson, S.J., and Abisgold, J.D. (1985). Compensation by locusts for changes in dietary nutrients: behavioural mechanisms. Physiol. Entomol. *10*, 443–452.

S4. Despland, E., and Simpson, S.J. (2005). Food choices of solitarious and gregarious locusts reflect cryptic and aposematic antipredator strategies. Anim. Behav. *69*, 471–479.

S5. Despland, E., and Simpson, S.J. (2005). Surviving the change to warning colouration: Density-dependent polyphenism suggests a route for the evolution of aposematism. Chemoecology *15*, 69–75.

S6. Simões, P., Ott, S.R., and Niven, J.E. (2011). Associative olfactory learning in the desert locust, *Schistocerca gregaria*. J. Exp. Biol. *214*, 2495-2503.

S7. Simpson, S.J., McCaffery, A.R., and Hägele, B.F. (1999). A behavioural analysis of phase change in the desert locust. Biol. Rev. *74*, 461–480.

S8. Roessingh, P., Simpson, S.J., and James, S. (1993). 104 Analysis of phase related changes in behavior of desert locust nymphs. Proc. R. Soc. B *252*, 43–49.

S9. Sokal, R., and Rohlf, F. (1998). Biometry: the Principles and Practice of Statistics in Biological Research (New York: W. H. Freeman and Company).
